# Hip Arthroscopy for Femoroacetabular Impingement Syndrome in High-Level Athletes: A 10-Year Follow-up

**DOI:** 10.1177/23259671241275657

**Published:** 2024-10-21

**Authors:** Louise Karlsson, Olle Collberg, David Erlandsson, Sarantos Nikou, Adad Baranto, Axel Öhlin, Ida Lindman

**Affiliations:** † Department of Orthopaedics, Institute of Clinical Sciences, Sahlgrenska Academy, University of Gothenburg, Gothenburg, Sweden; ‡ Department of Orthopaedics, Sahlgrenska University Hospital, Gothenburg, Sweden; § Department of Orthopaedic Surgery, South Älvsborg Hospital, Borås, Sweden; Investigation performed at Department of Orthopaedics, Sahlgrenska Academy, University of Gothenburg, and OrthoCenter, Gothenburg, Sweden

**Keywords:** hip, femoroacetabular impingement, hip arthroscopy, football (soccer), ice hockey, medical aspects of sports, athletes, patient-reported outcome measures

## Abstract

**Background:**

Arthroscopic treatment for femoroacetabular impingement syndrome (FAIS) has previously been reported to have favorable short-term results in high-level athletes. Less is known about long-term outcomes.

**Purpose:**

To report outcomes 10 years after hip arthroscopy for FAIS in high-level athletes using validated patient-reported outcome measures (PROMs).

**Study Design:**

Case series; Level of evidence, 4.

**Methods:**

Patients who underwent hip arthroscopy between November 2011 and January 2013 were included in a local hip arthroscopy registry and completed preoperative PROMs. At 10-year follow-up, the same PROMs were completed. Inclusion criteria were age <40 years at time of surgery, no prior hip surgeries, and a pre-symptomatic Hip Sports Activity Scale (HSAS) level of 7 or 8. The exclusion criterion was total hip arthroplasty at follow-up. The PROMs included the international Hip Outcome Tool-12 items (iHOT-12), the Copenhagen Hip and Groin Outcome Score (HAGOS), visual analog scale (VAS) for overall hip function, European Quality of Life-5 Dimensions questionnaire (EQ-5D) and European Quality of Life-visual analog scale (EQ-VAS), HSAS, and a single question on patient satisfaction. The rates of patients achieving minimal important change and reporting Patient Acceptable Symptom State (PASS) were reported for HAGOS and iHOT-12. For iHOT-12, preoperative results were compared with 1, 5, and 10-year follow-ups.

**Results:**

A total of 45 patients (34 men, 11 women; 70 hips; mean age 24.4 years at time of surgery) were included, with 77 patients eligible for inclusion. Significant improvements (*P* < .001) were seen at 10-year follow-up in all HAGOS subscales: Symptoms, Pain, Daily Activity, Sports, Physical Activity, Quality of Life (50.3 vs 78.6, 59.2 vs 86.8, 65.9 vs 88.8, 37.1 vs 81.1, 24.4 vs 81.1, 32.1 vs 79.3), iHOT-12 (40.1 vs 81.6), EQ-5D (0.59 vs 0.89), EQ-VAS (65.6 vs 80.4), and VAS for overall hip function (48 vs 79). For iHOT-12, the largest change was seen between preoperative and 1-year follow-up values, with consistent results over time. There was no statistically significant difference between HSAS levels preoperatively and at 10-year follow-up (HSAS level 4), with 24% of patients reporting a HSAS level 7 or 8 at the follow-up. Of the patients, 93% reported satisfaction with the surgery. PASS was achieved in 82% for iHOT-12, with a range of 76% to 91% for HAGOS subscales. Furthermore, 93% exceeded the minimal important change for iHOT-12, and a range of 67% to 84% for HAGOS.

**Conclusion:**

In a high-level athletic population, significant improvements in long-term outcomes are reported after hip arthroscopy for FAIS, with patients reporting a high satisfaction rate. The results also show that the largest improvement occurs within the first postoperative year, with results being maintained for 10 years.

Femoroacetabular impingement syndrome (FAIS) is common among young individuals and athletes.^15,16^ Sports imposing high demands on the hip joint, such as ice hockey and soccer, have previously been shown to be highly represented among patients with FAIS, as high repetitive loads on the hip during adolescence have been shown to increase the risk of developing an abnormal morphology of the joint.^1,3,28^ The morphological abnormalities of the joint, cam morphology on the femoral head and/or pincer morphology on the acetabulum, can lead to impingement during hip motion, increasing the risk for chondral damage.^27^ This can then lead to pain, stiffness, and reduced range of motion (ROM), especially in the athletic population.^1,28^

When treating FAIS surgically, arthroscopic treatment has become increasingly popular in the last decade, aiming to restore normal anatomy, reduce pain, and improve ROM. Several studies have previously reported on good short-term outcomes for surgical treatment.^2,9,14^ Furthermore, studies on athletes undergoing hip arthroscopy for FAIS have shown good short- and medium-term outcomes, with low rates of complications, reoperations, and few conversions to total hip arthroplasty (THA).^21,27^

Studies have recently started to emerge on long-term outcomes for general populations undergoing arthroscopic surgery for FAIS. These studies show improvements in hip function and quality of life when evaluating patient-reported outcome measures (PROMs) at 10-year follow-up.^5,6,18,22,23^ Previous studies on athletes have mostly evaluated return to sports after surgery.^7^ Although the rate of returning to sports is of major importance in this specific population, there is also a need to evaluate hip function and quality of life using PROMs, but only a few studies have been conducted on short-term outcomes.^11,19,21,29^ There is an even larger paucity in the literature on long-term follow-up in high-level athletes, especially using recommended hip-specific PROMs validated for a young and active population. Previous studies, both on athletic and general populations, have primarily evaluated treatment using PROMs developed for an elderly population with osteoarthritis.^4,6,8,17,22,23,31^

The primary aim of this study was to evaluate functional outcomes and quality of life 10 years after hip arthroscopy for FAIS in high-level athletes. The secondary aim was to evaluate functional outcomes over time compared with follow-up at 1 and 5 years postoperatively.

## Methods

### Study Design

All patients undergoing hip arthroscopy for FAIS between November 2011 and January 2013 were prospectively included in a local hip arthroscopy registry in Gothenburg, Sweden, and underwent surgery at 2 hospitals by 3 orthopaedic surgeons. Patients were asked to complete PROMs preoperatively and at 1, 5, and 10 years postoperatively. The PROMs consisted of the international Hip Outcome Tool-12 items (iHOT-12), the Copenhagen Hip and Groin Outcome Score (HAGOS), a visual analog scale (VAS) for overall hip function, European Quality of Life-5 Dimensions questionnaire (EQ-5D), and European Quality of Life-visual analog scale (EQ-VAS) for health-related quality of life. All PROMs were validated and translated into Swedish, with the hip-specific PROMs validated for a younger and active population.^13,25,26,30^ Postoperatively, the questionnaires included a single additional question on patient satisfaction on their surgery. Sports activity was measured by the Hip Sports Activity Scale (HSAS), ranging from 0 to 8, where a HSAS activity level of 7 or 8 describes a very high or elite level of sports participation.^25^ Table 1 provides further information on HSAS activity levels. Demographic and intraoperative data were reported by the treating orthopaedic surgeon. These included information on patient sex, age, and body mass index, and whether they had uni- or bilateral surgery, either at the same date or staged procedure within the inclusion period.

**Table 1:** Definition of HSAS Activity Levels^*a*^

HSAS Level	Description
0	No recreational or competitive sports
1	Recreational sports: swimming, cycling, hiking, Nordic walking (quick walking with ski poles)
2	Recreational sports: golf, bicycle riding, mountain biking, swimming, rowing, cross-country skiing/biathlon, dancing, inline skating
3	Recreational sports: aerobics, jogging, lower extremity weightlifting, horseback riding, cricket
4	Recreational sports: tennis, downhill skiing, snowboarding, indoor sports, baseball/softball
5	Competitive sports (minor leagues/collegiate): soccer, ice hockey, track and field, golf, bicycle racing, mountain biking, swimming, rowing, cross-country skiing/biathlon, horseback riding, cricket
6	Competitive sports (elite level): golf, bicycle racing, mountain biking, swimming, rowing, cross-country skiing/biathlon, horseback riding, cricket Competitive sports (minor leagues/collegiate): downhill skiing, snowboarding
7	Competitive sports (elite level): downhill skiing, snowboarding Competitive sports (minor leagues/collegiate): soccer, ice hockey, field hockey, American football, rugby, martial arts, tennis, track and field, indoor sports, beach volleyball, lacrosse, baseball/softball
8	Competitive sports (elite level): soccer, ice hockey, field hockey, American football, rugby, martial arts, tennis, track and field, indoor sports, beach volleyball, lacrosse, baseball/softball

a HSAS, Hip Sports and Activity Scale.

The surgical technique has previously been described.^27^ In summary, the hip arthroscopy was individualized for all patients based on history, radiology, clinical, and intraoperative findings. An anterolateral and a midanterior portal were used. To examine the central compartment, axial traction was applied. A ligament-sparing capsulotomy was used to reach the peripheral compartment. The surgical approach did not include capsular closure, since no transverse capsulotomy was performed. Pincer deformities were resected with an “over the top” technique, with the bur placed in the perilabral sulcus. In the event of small pincer resections, the labrum was left in situ. With larger deformities with a clear separation between the labrum and the acetabular edge, the labrum was reattached with suture anchors. All femoral abnormalities were carefully resected. To accomplish this, resection occurred at all the accessible parts of the femoral neck. Resection of pistol-grip deformities reached far posterior to the lateral retinacular fold, with careful attention paid to avoid damage to the lateral retinacular vessels, which could result in osteonecrosis of the femoral head. Perioperative fluoroscopy was continually used to control the extent of the bony resection. Patients were permitted free ROM directly after the surgery, with full weightbearing allowed, although crutches were recommended for outdoor walking in the first 4 weeks after surgery. Physiotherapy was initiated immediately postoperatively, and the intensity of the rehabilitation was progressively amplified in accordance with the patient and symptoms. The protocol comprised exercises for strength, endurance, stability, coordination, and ROM. To avoid heterotopic ossification, the patients were prescribed nonsteroidal anti-inflammatory drugs for the first month postoperatively.

Inclusion criteria were patients aged under 40 years at the time of surgery, who had undergone primary surgery for FAIS, and had a presymptomatic HSAS level of 7 or 8. The exclusion criterion was conversion to THA during the 10-year follow-up. The questionnaires were sent out between 120 and 136 months postoperatively by email. Those who did not respond were then contacted by telephone and were asked to complete the questionnaire. The present study was approved by the Regional Ethical Review Board in Gothenburg, Sweden (ID No.: 071-12).

### Statistical Analysis

The data analyses were performed using SPSS version 29.0 (IBM Corp). Descriptive statistics were used for demographic variables and presented as mean, standard deviation, median, and range. Nominal data were reported as numbers and percentages. For comparisons of PROMs preoperatively and at the 10-year follow-up, the Wilcoxon signed-rank test was used. A power analysis was conducted for previous follow-ups on this population and enrollment undertaken accordingly, but not for this specific 10-year follow-up. However, a post-hoc analysis was carried out on iHOT-12, which gave a power of 91%. Significance was set at *P* < .05.

To evaluate the outcome regarding the minimal important change (MIC), the number of patients exceeding the MIC was reported. A distribution-based technique was used to determine the MIC, at 0.5 times the standard deviation of the change in the score. To evaluate the Patient Acceptable Symptom State (PASS), previously established results were used for cutoff values for iHOT-12 and HAGOS, with a threshold for iHOT-12 at 63.0.^24^ For HAGOS, the thresholds for PASS were as follows: 62.5 for symptoms; 68.8 for pain; 82.5 for function of daily living; 60.9 for sports; 43.8 for physical activity; and 42.5 for quality of life.^12^ To examine changes over time in mean iHOT-12 values, a repeated measures analysis of variance calculation was carried out with pairwise comparisons between time points.

## Results

### Patients and Clinical Characteristics

**Figure 1.**
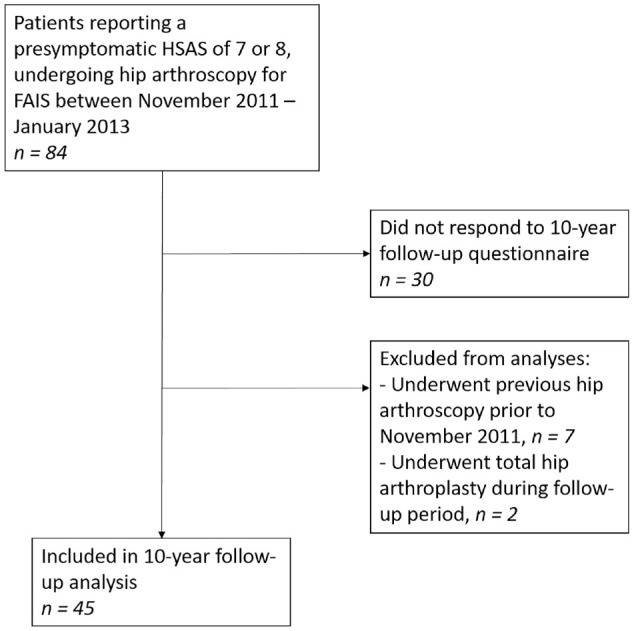
Flowchart of included patients. FAIS, femoroacetabular impingement syndrome; HSAS, Hip Sports Activity Level; PROMs, patient-reported outcome measures.

During the study period (November 2011 to January 2013), 84 patients with a presymptomatic HSAS of 7 or 8 underwent hip arthroscopic surgery for FAIS. After reviewing the medical charts, a total of 2 patients were excluded from the study as a result of receiving a THA, and 7 patients were found to have undergone previous hip arthroscopy before the study inclusion period and were therefore also excluded (Figure 1). Finally, a total of 45 patients who underwent arthroscopic surgery for FAIS were included in the present study, resulting in a 36% loss to follow-up.

The population consisted of 34 men and 11 women, with a total number of 70 included hips, as 25 patients underwent bilateral surgery (Table 2). The mean age (±SD) at the time of surgery was 24.4 ± 6.3 years with a mean symptom duration of 33.2 ± 40.8 months before surgery. A detailed list of the surgical procedures is summarized in Table 2. Of the 77 patients eligible for inclusion in the present study, 5 (6.5%) patients underwent reoperation due to FAIS during the 10-year follow-up period, where all patients underwent a revision of their cam resection. The mean follow-up time for the present study was 129 ± 6.1 months. A detailed list of the performed sports is found in Table 3.

**Table 2:** Patient Demographics and Perioperative Data^*a*^

Demographics and Perioperative Data	Total
Patients, n	45
Hips, n	70
Side of surgery, n (%)	
Right	8 (18)
Left	12 (27)
Bilateral	25 (56)
Sex, n (%)	
Male	34 (76)
Female	11 (24)
Age at time of surgery, y (mean ± SD)	24.4 (±6.3)
BMI, kg/m^2^-(mean ± SD)	23.8 (±2.1)
Symptom duration, mo (mean ± SD) (n=42)	33.2 (±40.8)
Follow-up time, mo (mean ± SD)	129 (±6.1)
Type of surgery	
Cam resection, n	37
Pincer resection, n	0
Combined cam + pincer, n	31
Missing information, n	2
Additional procedure	
Labral resection, n	9
Labral suture, n	3
Microfracture, n	4

a BMI, body mass index; L, left; mo, months; n, number; R, right.

**Table 3:** Types of Sports Performed

Sport Performed	Number (%)
Soccer	28 (62)
Ice hockey	5 (11)
Handball	3 (7)
Martial arts	3 (7)
Figure skating	2 (4)
Dance	1 (2)
Basketball	1 (2)
Bandy	1 (2)
American football	1 (2)

### PROMs

A comparison between the PROMs at the 10-year follow-up and preoperative values showed statistically significant improvements in all HAGOS subscales, along with iHOT-12, EQ-5D, EQ-VAS, as well as VAS for overall hip function (Table 4). Of the patients, 93% reported satisfaction with the surgery. The mean change in HSAS level compared with their preoperative level was not significant (Table 5). A total of 11 (24%) patients reported a HSAS level of 7 or 8 at the 10-year follow-up. In the HAGOS subscales, MIC values were exceeded by 82% for the Symptom subscale, 82% for Pain, 67% for Activities of Daily Living, 84% for Sport/Recreation, 84% for Physical Activity, and 84% for Quality of Life. When reviewing iHOT-12, a total of 93% exceeded MIC values. Between 76% and 91% reported PASS in all HAGOS subscales. For iHOT-12, 82% of the patient group achieved PASS (Table 6).

**Table 4:** Patient-Reported Outcome Data^*a*^

Outcome Measure	Preoperative	10 Years	Δ	Δ^*b*^	*P* Value
HAGOS					
Symptoms	50.3 ± 18.6	78.6 ± 18.3	28.3 ± 20.6	28.6 (-28.6-64.3)	**<.001**
Pain	59.2 ± 16.9	86.8 ± 18.2	27.7 ± 18.7	30.0 (-17.5-67.5)	**<.001**
Daily activity	65.9 ± 21.5	88.8 ± 19.4	22.9 ± 21.6	25.0 (-20.0-80.0)	**<.001**
Sports	37.1 ± 19.2	81.1 ± 22.4	44.0 ± 25.2	40.6 (-15.6-90.6)	**<.001**
Physical activity	24.4 ± 24.6	80.8 ± 25.5	56.4 ± 34.6	62.5 (-25-100.0)	**<.001**
Quality of life	32.1 ± 20.6	79.3 ± 25.0	47.2 ± 27.0	45.0 (-10.0-95.0)	**<.001**
iHOT-12	40.1 ± 15.9	81.6 ± 20.4	41.5 ± 20.8	42.2 (-2.1-75.7)	**<.001**
EQ-5D	0.59 ± 0.27	0.89 ± 0.17	0.30 ± 0.25	0.28 (-0.07-0.77)	**<.001**
EQ-VAS	65.6 ± 17.4	80.4 ± 17.9	14.8 ± 19.0	10.0 (-20.0-70.0)	**<.001**
VAS—hip function	48.2 ± 19.8	79.2 ± 21.0	31.0 ± 24.4	35.0 (-45.0-100.0)	**<.001**
Satisfaction, n (%)	NA	42 (93)	NA	NA	NA

a Data are presented as mean ± SD unless otherwise indicated. Δ, change 10 years - preoperative; EQ-5D, European Quality of life 5-Dimensions questionnaire; EQ-VAS, European Quality of life-visual analog scale; HAGOS, Copenhagen Hip and Groin Outcome Score; HSAS, Hip Sports and Activity Scale; iHOT-12, international Hip Outcome Tool-12 items; NA, not applicable; Satisfaction, satisfied with surgery at 10 years; VAS, visual analog scale. All significant p-values are written in bold.

b Data are presented as median (range).

**Table 5:** HSAS Responses Preoperative and 10-Years Postoperative (N = 38)^*a*^

HSAS Level	Preoperative	10-Year Follow-up	Δ^*b*^	*P* Value
8	6 (16)	5 (13)	NA	NA
7	5 (13)	6 (16)	NA	NA
6	3 (8)	2 (5)	NA	NA
5	4 (11)	3 (8)	NA	NA
4	6 (16)	8 (21)	NA	NA
3	4 (11)	8 (21)	NA	NA
2	6 (16)	4 (11)	NA	NA
1	2 (5)	1 (3)	NA	NA
0	2 (5)	1 (3)	NA	NA
Total	38 (100)	38 (100)	NA	NA
Median	4	4	NA	NA
HSAS ≥5	18 (47)	16 (42)	NA	NA
Mean ± SD	4.50 ± 2.5	4.58 ± 2.2	0.08 ± 2.84	.985

a Data are presented as n (%) unless otherwise indicated. Δ, change from preoperative to 10 years postoperative; HSAS, Hip Sports Activity Scale; NA, not applicable.

b Data are presented as mean ± SD.

**Table 6:** Numbers and Percentages of Patients Exceeding MIC and Reporting PASS^*a*^

PROMs	Exceeded MIC	Reported PASS
HAGOS		
Symptoms	37 (82.2)	35 (77.8)
Pain	37 (82.2)	38 (84.4)
Activities of Daily Living	30 (66.7)	34 (75.6)
Sports and Recreation	38 (84.4)	36 (80.0)
Physical Activity	38 (84.4)	41 (91.1)
Quality of Life	38 (84.4)	40 (88.9)
iHOT-12	42 (93.3)	37 (82.2)

a Data are presented as n (%). HAGOS, Copenhagen Hip and Groin Outcome Score; iHOT-12, international Hip Outcome Tool-12 items; MIC, minimal important change; MCID, minimal clinically important difference; PASS, Patient Acceptable Symptom State; PROMs, patient-reported outcome measures.

**Figure 2.**
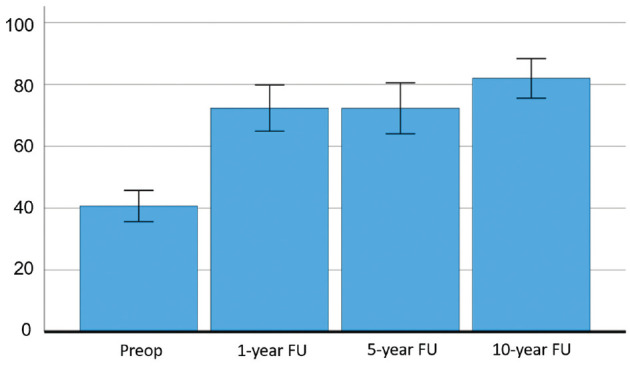
Progression of mean iHOT-12 score from preoperative values to 10-year follow-up, with additional plot showing 95% CI, n = 42. FU, follow-up; iHOT-12, international Hip Outcome Tool-12 items.

When comparing the 42 patients who completed all iHOT-12 scores preoperatively and at the 1, 5, and 10-year follow-ups, the mean values shown in the present study were similar at 1 and 5 years and significantly slightly higher at 10 years (Figure 2). The largest improvement in mean values occurred between the preoperative score and the 1-year follow-up (mean change 31.6; *P* < .001). No statistically significant change was seen between 1 and 5 years (mean change 0.063; *P* >.999). However, between the 5- and 10-year follow-ups, a significant change was seen, with a higher mean iHOT-12 at the 10-year follow-up (mean change 9.7; *P* = .011).

## Discussion

The present study shows a statistically significant improvement in outcomes 10 years after hip arthroscopy for FAIS, relating to both hip function and quality of life compared with preoperative PROM values, with >9 out of 10 patients reporting satisfaction with the surgery. Most of the patients reported a clinically relevant improvement, especially in relation to physical activity, reduced pain, and quality of life. Moreover, an improvement was reported for all included PROMs, except for HSAS where no significant change was seen between preoperative values compared with the 10-year follow-up. Comparing PROM results preoperatively and at the 1-, 5-, and 10-year follow-ups showed a clear significant short-term improvement that stayed consistent in the long term, indicating good postoperative results that were maintained for a long time in a young, athletic population.

Both 1- and 5-year follow-up outcomes for the athletes evaluated in this study have previously been published.^21,27^ The high survivorship rate further indicates that FAIS surgery is a safe method in the long term. The 5-year follow-up reported that 54% of all patients still participated in competitive sports (HSAS levels 5-8), with 77% of all patients reporting a decrease in HSAS levels compared with their HSAS levels before symptom onset. In the 5-year study, it was also shown that the patients reporting a decrease in HSAS had longer symptom durations preoperatively and were older at the time of surgery. It should be expected that patients report a decrease in their HSAS levels at the 10-year follow-up, due to other factors than just their hip function after FAIS surgery, as the probability of retiring from a high level of sports increases with age. Interestingly, 29% of the cohort reported a HSAS level of 7 or 8 in the present study, same as the percentage reported preoperatively. Hence, due to a long follow-up, where patients may show a natural decline in activity level, the HSAS should not be considered a reliable tool in assessing the long-term results of surgery in this study.

A recent systematic review of 12 studies reported outcomes on long-term follow-up (range, 10-20 years) after hip arthroscopy, with cohort sizes ranging from 38 to 384 included hips.^18^ Of these studies, 10 reported on PROMs, with 8 studies reporting a significant improvement in at least one of the included PROMs. The reoperation rates ranged from 5% to 24% and the failure rate, ie, conversion to THA, was in the range of 0% to 44%. None of the included studies used the same PROMs as the present study, including HSAS. The included studies used PROMs not validated for younger athletic populations and whose use has been discouraged for the evaluation of outcomes in FAIS populations due to lack of content validity.^10^ The hip-specific PROMs used in the present study, HAGOS and iHOT-12, have been validated for an active population, making them more suitable to use for high-level athletic patients.^13,25,30^ Hopefully, in the future, a universally agreed hip-specific PROM for FAIS will be agreed upon.

The included patients in the systematic review did not all undergo surgery for FAIS, with some studies only including labral tears, “labral disorders,” synovitis, avascular necrosis, capsular laxity, and dysplasia.^18^ Although the present study only included patients with a diagnosis of FAIS, a relatively small percentage also underwent additional labral debridement or repair. Compared with the mean patient age of 24 years in the present study, the cohorts in the systematic review also included older patients (mean age, 32-44 years) and 1 study only focused on adolescents (mean age, 16 years). Moreover, all 5 studies that included MIC and PASS had at least 1 PROM where 80% achieved a MIC difference, and at least 1 PROM where 75% of the cohort reached an acceptable symptom state, which is comparable with or inferior to what was found in the present study. However, the present study had a more active population, which could bias the PASS results, as the threshold numbers were taken from a study on outcomes in a general population.^12,24^

In a previous study, where PASS values were developed and calculated for all HAGOS subscales, a total of 137 patients who underwent hip arthroscopy for FAIS were included and followed up at 18 months.^12^ At this follow-up, 47% achieved PASS, lower than the results found in the present study even though the follow-up time was shorter. However, that study used a general population, with a higher mean age (35 years) and lower mean scores in all HAGOS subscales, potentially explaining the discordant results compared with this study.

### Strengths and Limitations

To the best of the authors’ knowledge, the present study is the first to report on long-term outcomes for hip arthroscopy in a high-level athletic population with FAIS using validated PROMs appropriate for this population. Many patients also completed questionnaires at all follow-ups, making it possible to compare results at several postoperative time points. All patients included in this study also had a thorough medical chart review undertaken to investigate possible previous surgeries, reoperations, and THA surgeries. However, there is a possibility that some patients could have undergone either reoperation or THA at other hospitals in Sweden that could have been missed. Another strength of this study is the high external validity of the results, as a previous study on loss to follow-up in the register concluded that PROM results from the registry were generalizable.^20^

The most important limitation in this study was the relatively low response rate at follow-up. This renders both a risk for selection bias and type 2 error. Patients were also included if they had a HSAS of 7 or 8 before symptom onset, not at the time of surgery. As symptom duration varied greatly, there is a risk that some patients had already retired from high-level sports at the time of surgery with no plan of returning to a HSAS level of 7 or 8. There is also a risk of recall bias, as patients could be reporting an incorrect activity level if a long time had passed between symptom onset and time of surgery. Differences in surgical technique in Sweden compared to other countries as it pertains to labral debridement/repair can also make the results of the present study less generalizable. However, previous studies from the Swedish hip arthroscopy registry have reported similar outcomes as the Danish hip arthroscopy registry, where labral debridement/repair is standard, thus making the results from the present study more generalizable.^27,11^

## Conclusion

In a high-level athletic population, significant improvements in long-term outcomes are reported after hip arthroscopy for FAIS, with patients reporting a high satisfaction rate. The results also show that the largest improvement occurs within the first postoperative year, with results being maintained for 10 years.
